# Homes for Incurables

**Published:** 1887-01-22

**Authors:** Louisa Twining


### Homes for Incurables: Free Payments *v.* The Voting System.

By Miss Louisa Twining.

May I say a word on the subject of incurables, in which class I have been long interested, as your columns invite discussion? I was acquainted with the founder (as I think he was) of the British Home for Incurables, Mr. Charles Hood, and about the same time I was starting a Home to help such cases, for which he kindly gave me a generous gift. Had I been rather sooner in the field, I believe he would have co-operated in my scheme still further; and not many years since I discussed the whole subject with him. The two Homes have now accomplished their quarter of a century, the only previous effort being, I believe, that at Highbury. The two chief points on which our plans differed were, our receiving payments and avoiding the voting system. I will not enter now upon the latter question (though I may express a hope that you will some day allow a discussion on this most important subject, especially as it regards the sick and aged), but I may say that we have survived and flourished without it, and by merely entering the names of applicants, who *knew* they must wait their turn for admission, and were received as vacancies occurred.

With regard to the other point of annual payments, I have never been able to understand why these should be refused by the British Home and by the Hospital for Incurables. We have had hundreds of applicants, (over 100 in the year), but none have asked to come "free"; we have invariably found that they either had pensions of £20 or so (very often from City companies), or had relations and friends able and willing to pay such sums, in order to ensure the care and nursing which *they* could not give; and why sons and daughters (often employed away from home), and employers who have been faithfully served in various capacities, should not be allowed to help in supporting the aged and incurable, I have never been able to understand.

Poor-law Infirmaries are now being made suitable asylums for destitute incurables, but the class above them who may have savings, pensions, or friends, I think should be encouraged to pay at least what they can towards their maintenance, and not be classed as "paupers," or living on charity. I cannot but think it is a great mistake to encourage any class of persons to cast off friends and relations, who have a claim upon them, to the care of strangers and the public.

